# Goal setting in stroke rehabilitation: A systematic review and qualitative synthesis of the experiences and perspectives of healthcare professionals

**DOI:** 10.1177/02692155261446313

**Published:** 2026-05-02

**Authors:** Anna Fišerová, Šarlota Duchoňová, Hannah Hafiz, Martina Hagarová, Brodie Morton, Helena Tessmann, Christopher D. Graham, Nils Rickardsson

**Affiliations:** 1 Department of Psychological Sciences and Health, 3527 University of Strathclyde, Glasgow, UK; 2 Oxford Institute of Clinical Psychology Training and Research, 6396 University of Oxford, Oxford, UK

**Keywords:** Goal setting, stroke rehabilitation, healthcare professional, person-centred, experiences

## Abstract

**Objective:**

To synthesise and analyse qualitative evidence relevant to the question: What are the experiences and perspectives of healthcare professionals on goal setting in stroke rehabilitation?

**Data sources:**

PubMed, PsycINFO, MEDLINE and CINAHL were systematically searched in May 2025, supplemented by backward and forward citation searching.

**Review methods:**

This systematic review was pre-registered on PROSPERO (CRD420251038210). Eligibility criteria included peer-reviewed qualitative or mixed methods studies with qualitative data from healthcare professionals outlining experiences of goal setting in stroke rehabilitation. Non-English publications were excluded. The Critical Appraisal Skills Programme (CASP) checklist was used to appraise quality. Data were analysed using thematic synthesis.

**Results:**

Eight studies, published between 1999 and 2020, were included. These comprised 108 clinicians of various rehabilitation disciplines, from multiple countries, working across acute, inpatient and community settings. Most data were collected via semi-structured interviews. Methodological rigour of identified studies was generally high. Nine descriptive themes emerged from the thematic synthesis. From these descriptive themes, three analytical themes were derived: (1) Who leads, who follows?, (2) Between hope and reality, (3) Starting with the person, not the problem. Eight of the descriptive themes were directly related to analytical themes, whereas one theme was a stand-alone theme. Confidence in the thematic synthesis findings was assessed as moderate.

**Conclusion:**

This synthesis of qualitative studies from various rehabilitation settings in stroke found that experiences of goal setting from the perspective of healthcare professionals describe directive and collaborative approaches, emotional aspects of goal setting in time-limited contexts and a commitment to person-centred care.

## Introduction

Goal setting is an integral component of stroke rehabilitation. It refers to a collaborative process where patients and one or several members of the multidisciplinary team agree on specific, often time-limited, targets.^1^ Such collaboration helps ensure that goals are both personally meaningful to the patient and clinically focused in light of stroke-related impairments. In practice, goals in rehabilitation may involve short-term, quantifiable aspects of progress in the rehabilitation setting, such as walking a twenty-metre distance for someone with mobility problems. Goals can also be more long-term and relate to participation in the community, such as attending a concert with a loved one.

Research suggests that early and active involvement of stroke survivors and their caregivers in goal setting can enhance motivation, engagement, autonomy and satisfaction, as well as improve functional outcomes.^2,3^ Given these benefits, it is unsurprising that collaborative goal setting is strongly advocated in stroke guidelines.^4,5^ Despite this, guidelines are not routinely implemented in clinical practice.^1^

Systematic review findings have identified common patient experiences that highlight aspects of rehabilitation that impact on participation in goal setting, including cognitive and communication difficulties, logistics and conflicting expectations between patients, staff and family members.^1,6,7^ Less is known about these experiences from the perspective of health care professionals, who have a central role in initiating goal setting. Given the significant and long-term impact of stroke on individuals, families, health services and society at large, and the promise that goal setting holds for improving rehabilitation outcomes, gaining a deeper understanding of clinicians’ experiences is essential.

Qualitative systematic reviews have provided in-depth insights into aspects of stroke rehabilitation, such as the patient experience of goal setting and perspectives of healthcare professionals when implementing clinical guidelines.^8,9^ Individual qualitative studies have examined the experience of healthcare professionals delivering goal setting. To date, no qualitative review has brought together this body of research to derive a broader understanding of stroke healthcare professionals’ perspectives in facilitating goal setting, despite their central role in this process.

Our aim was to explore health care professional's experiences and perspectives, generally including, for example, challenges, barriers, benefits and views on goal setting in stroke rehabilitation settings. We sought out to synthesise the existing literature relevant to the research question: What are the experiences and perspectives of healthcare professionals on goal setting in stroke rehabilitation?

## Methodology

This study adopted a systematic review design with a qualitative synthesis. To enhance transparency and methodological rigour, the study adhered to PRISMA^10^ and ENTREQ^11^ guidelines. The review was pre-registered on PROSPERO (CRD420251038210).

A systematic search to identify relevant studies was undertaken and reported using a PRISMA flow diagram.^10^ Four electronic databases (PubMed, PsycINFO, MEDLINE and CINAHL) were systematically searched in May 2025 with no restrictions on publication date.

The SPIDER tool (Sample, Phenomenon of Interest, Design, Evaluation, Research type)^12^ was used to create search terms (see Supplemental file, Table 1) and criteria for inclusion. The inclusion and exclusion criteria comprised any professional working within stroke rehabilitation, and studies using qualitative methods of exploring experiences of goal setting for this population. Studies were excluded if quotes could not be separated or identified as coming from healthcare professionals (full inclusion/exclusion criteria are available in Supplemental Table 2).

The titles and abstracts of all retrieved studies were uploaded to EndNote. After removing duplicates, titles and abstracts were screened against the inclusion and exclusion criteria by AF. Full texts of studies were then screened independently for eligibility by several members of the research group (ŠD, AF, HH, MH and BM), and final inclusion decisions were agreed collaboratively following discussion. Backward and forward citation searching were used to cross-reference all included papers.

The Critical Appraisal Skills Programme (CASP) Checklist^13^ was employed to evaluate the methodological quality of included studies. In line with GRADE-CERQual guidelines,^14^ the CASP ratings were used to contextualise results by highlighting strengths and limitations of included studies (and confidence in themes), rather than serving as exclusion criteria. All included studies were assessed (with CASP checklist) independently by several members of the research group (ŠD, AF, HH, MH and BM), and a Fleiss’ kappa of .91 was achieved, with final rating agreed through discussion.

Data extracted from each included study comprised the title, authors, year and country of publication, study aims, number of clinician participants, clinicians’ professional backgrounds, setting, study methodology and data collection methods. Additionally, clinicians’ quotes from the results sections were extracted for the purpose of thematic synthesis.

Data were analysed using the thematic synthesis method described by Thomas and Harden.^15^ Thematic synthesis in this study followed a three-stage process. In Stage 1, data from the included studies were compiled into a Word document and coded line by line. In Stage 2, descriptive themes were developed by organising related codes into broader categories that captured shared patterns across the data. In Stage 3, these descriptive themes were further interpreted and refined into higher-order analytical themes, offering new insights that moved beyond the content of the original studies.

Stage 1 and Stage 2 of the analysis were completed by one researcher (AF). During Stage 3, the themes were discussed and refined iteratively through discussions with additional members of the research group (CDG and NR) with clinical experience of goal setting in stroke rehabilitation. This process allowed reflexivity and additional perspectives as part of the development of the analytical themes from the data.

Following this, the confidence in the findings from the data synthesis was assessed using the GRADE CERQual guidelines.^14^ The approach provides a transparent and systematic way to assess the extent to which the findings accurately represent the phenomenon of interest, thereby enhancing the validity and reliability of the review's results.

## Results

The PRISMA flowchart (Figure 1) demonstrates the study selection process. Five members of the research group assessed full texts independently (ŠD, AF, HH, MH and BM), with discrepancies discussed until consensus was reached. Fleiss’ kappa was 0.77 before discussion and 1.0 after consensus.

**Figure 1.**
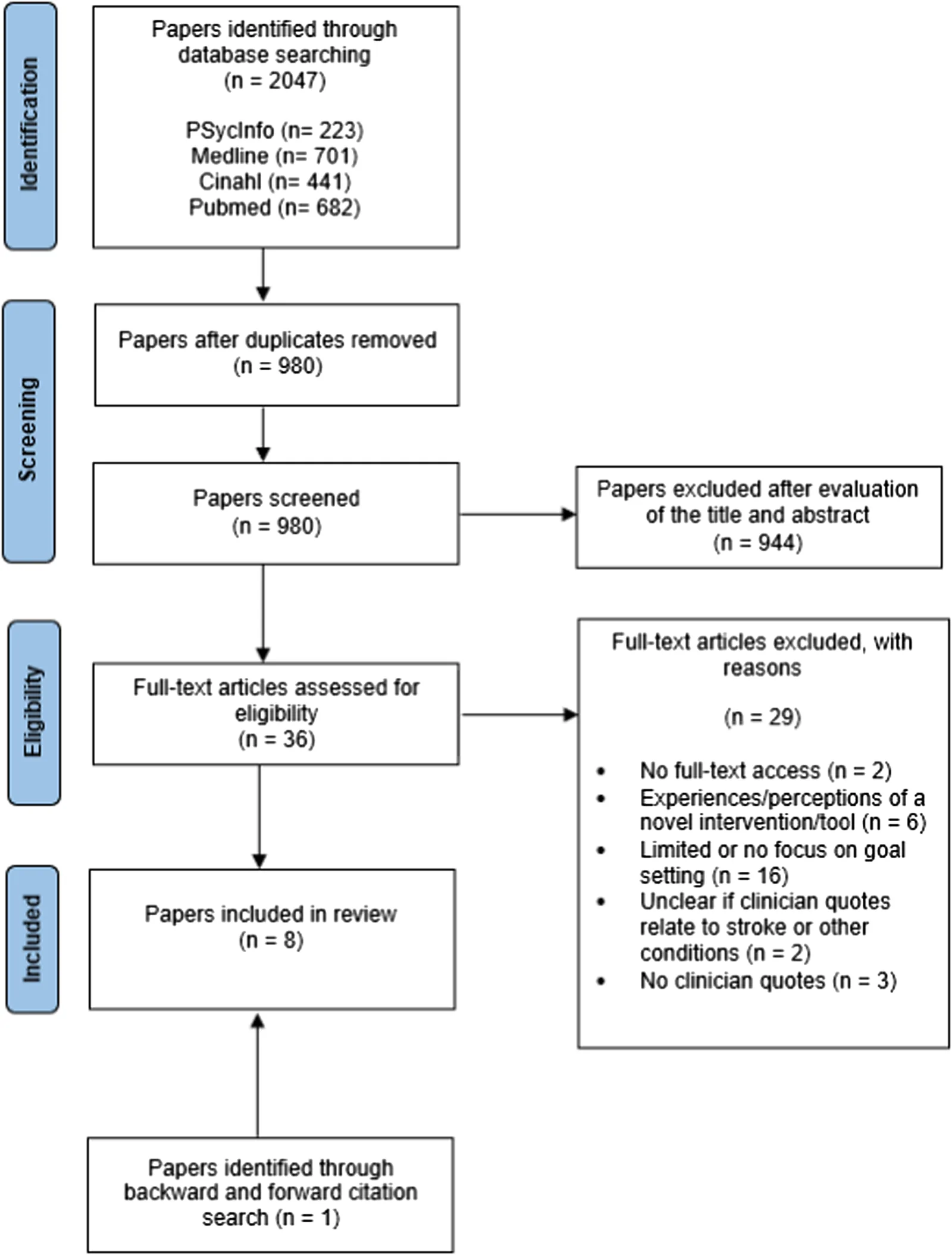
PRISMA flowchart.

As shown in Figure 1 and Table 1, eight studies were included in the review. The most common reason for exclusion from full-text screening was lack of focus on goal setting as a process of exploration in studies. Publication dates ranged from 1999 to 2020. Four studies were from the United Kingdom and one from each of the following countries: Austria, Australia, New Zealand and Norway. Altogether, the studies included 108 healthcare professionals. Those included physiotherapist (*n* = 24), speech pathologists (*n* = 22), occupational therapists (*n* = 19), nurses (*n* = 18), speech and language therapists (*n* = 13), physicians (*n* = 6), psychologists (*n* = 2), social workers (*n* = 2), cultural advisor (*n* = 1) and senior house officer (*n* = 1). Most studies collected data via semi-structured interviews.

**Table 1.** Summary of included studies.

Study	Country	Aim	*N* of clinicians	Professional background	Setting	Study methodology and data collection
Berg et al.^16^	Norway	To explore speech pathologists’ experiences of client participation in goal setting and clinical decision making for individuals with stroke-induced aphasia.	20	Speech pathologists (*n* = 20)	Acute, inpatient and outpatient rehabilitation	Recruited via professional networks and conference lists; semi-structured focus groups
Dörfler and Kulnik^17^	Austria	To understand clinicians’ practice-based strategies for involving stroke survivors with communication and/or cognitive impairment in person-centred goal setting.	11	Occupational therapists (*n* = 5), physiotherapist (*n* = 1), psychologists (*n* = 2), speech and language therapists (*n* = 3)	Acute, inpatient and outpatient rehabilitation	Purposive snowball sampling; semi-structured interviews (in person/remote)
Lawler et al.^18^	England	To explore how specialist nurses used goal setting in practice and how it shaped their ongoing relationships with stroke patients, including its perceived value and concerns raised by patients and caregivers during the first year post-stroke.	5	Specialist nurses (*n* = 5)	Community-based outpatient rehabilitation	Recruited from Elderly Care department; semi-structured interviews
Leach et al.^19^	Australian	To examine therapists’ perspectives on current goal setting practices in a subacute rehabilitation setting.	8	Occupational therapists (*n* = 3), Speech pathologists (*n* = 2) and physiotherapists (*n* = 3)	Inpatient rehabilitation	Recruited from Geriatric Assessment and Rehabilitation Unit; semi-structured email interviews using case examples
Levack et al.^20^	New Zealand	To investigate how goal setting is applied in inpatient stroke rehabilitation.	28	Physicians (*n* = 6), nurses (*n* = 11), physiotherapists (*n* = 4), occupational therapists (*n* = 3), social workers (*n* = 2), speech and language therapist (*n* = 1), cultural advisor (*n* = 1).	Inpatient rehabilitation	Recruited from public hospitals; semi-structured interviews, observation on the ward and informal discussion about the case
Lloyd et al.^21^	England	To describe physiotherapists’ views and experience on collaborative goal setting with stroke patients in sub-acute hospital care.	9	Physiotherapists (*n* = 9)	Acute and inpatient rehabilitation	Recruited from NHS hospitals; semi-structured interviews
Parsons et al.^22^	England	To explore clinicians’ views on factors affecting stroke survivors’ active engagement in goal setting.	20	Occupational therapists (*n* = 7), physiotherapist (*n* = 5), speech and language therapists (*n* = 8)	Acute rehabilitation, community based rehabilitation	Recruited from stroke rehabilitation teams; semi-structured interviews
Rosewilliam et al.^23^	England	To examine the patient-centredness of goal setting in acute stroke rehabilitation, and identify factors affecting its implementation.	7	Occupational therapist (*n* = 1), physiotherapists (*n* = 2), nurses (*n* = 2), speech and language therapist (*n* = 1), Senior house officer (*n* = 1)	Acute rehabilitation	Purposive sampling from a hospital; semi-structured interviews

Table 2 shows that the overall quality ratings were high, except for Lawler et al.^18^ In two other studies, it was unclear whether the researcher–participant relationship was adequately considered (CASP Criterion 6), which is particularly important given their use of semi-structured interviews.

**Table 2.** Critical appraisal skills programme (CASP) quality assessment.

Study	Criteria									
	1^a^	2^b^	3^c^	4^d^	5^e^	6^f^	7^g^	8^h^	9^i^	10^j^
Berg et al.^16^	✓	✓	✓	✓	✓	✓	✓	✓	✓	✓
Dörfler and Kulnik^17^	✓	✓	✓	✓	✓	✓	✓	✓	✓	✓
Lawler et al.^18^	✓	✓	✓	—	—	**X**	**X**	—	✓	✓
Leach et al.^19^	✓	✓	✓	✓	✓	—	✓	✓	✓	✓
Levack et al.^20^	✓	✓	✓	✓	✓	✓	✓	✓	✓	✓
Lloyd et al.^21^	✓	✓	✓	✓	✓	✓	✓	✓	✓	✓
Parsons et al.^22^	✓	✓	✓	✓	✓	—	✓	✓	✓	✓
Rosewilliam et al.^23^	✓	✓	✓	✓	✓	✓	✓	✓	✓	✓

✓: Yes; **X**: No; —: Can’t tell.

a Research aims clear. ^b^Qualitative methodology appropriate. ^c^Design appropriate to aims. ^d^Appropriate recruitment strategy. ^e^Data collection appropriate. ^f^Adequate consideration of researcher-participant relationships. ^g^Adequate consideration of ethical issues. ^h^Data analysis rigorous. ^i^Clear statement of findings. ^j^Valuable research.

### Thematic synthesis

The thematic synthesis identified nine descriptive themes, from which three analytical themes were developed. These are presented in Figure 2 to illustrate their relationships. The descriptive themes capture specific aspects of clinicians’ experiences, which are brought together within the analytical themes to represent broader patterns in the data. One descriptive theme, ‘Emotions shape the conversation’, is presented as a standalone theme, as it represents a distinct aspect of clinicians’ accounts not integrated within the analytical themes. The titles of the themes reflect the perspective of healthcare professionals and are grounded in participant language and quotes. Additional supporting quotes for themes are provided in Supplemental Table 4.

**Figure 2.**
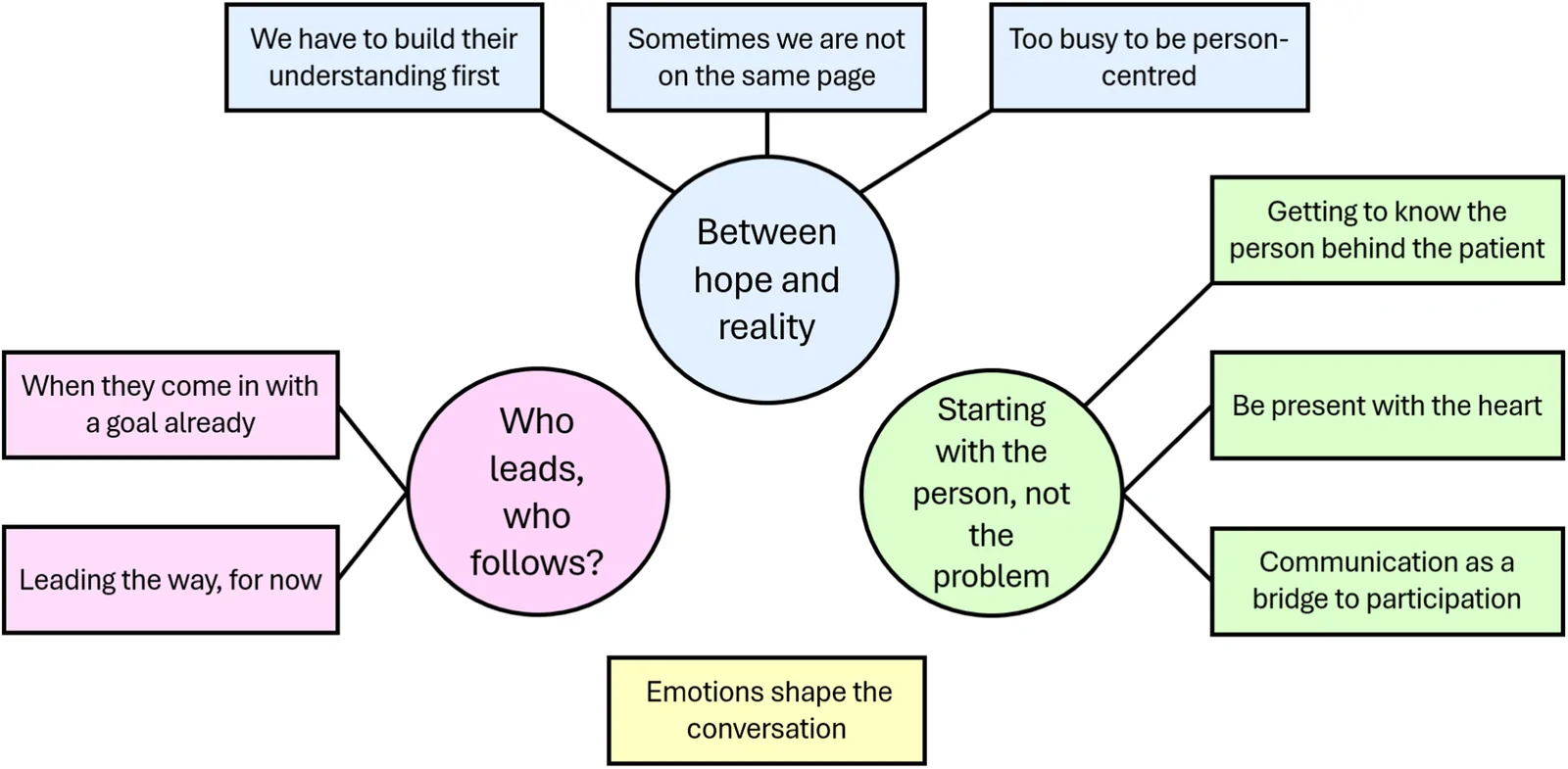
Relationships between descriptive and analytical themes.

Note: Analytical themes are presented in circles and descriptive themes in rectangular boxes. Lines indicate which analytical theme each descriptive theme contributes to. Eight descriptive themes contribute to three analytical themes, while ‘Emotions shape the conversation’ is presented as a standalone descriptive theme.

### Descriptive themes

The nine descriptive themes identified are outlined below and illustrated within the rectangular boxes in Figure 2. The individual studies contributing to each descriptive theme are listed in Supplemental Table 3.

### When they come in with a goal already

Clinicians observed that some patients arrived at rehabilitation with clear, well-articulated goals, which facilitated a smoother and more person-centred approach to rehabilitation planning. One practitioner noted that such patients ‘*pretty much tell you what they want […] before you even get to asking’*^20^ (p. 211). Another clinician similarly reflected, ‘*If he or she is able to participate it is of course much easier to make the goals oriented towards what the client wants’*^16^ (p. 874). This readiness allowed clinicians to align treatment plans directly with patient aspirations, making goal setting structured and collaborative.

### Leading the way, for now

Healthcare professionals also described taking a directive role in goal setting initially, based on their perception of patient needs. For example, one physiotherapist explained, ‘*The goal setting process was controlled by myself. After assessment of the patient, I determined what impairments she had and then decided to treat those’*^19^ (p. 163). In some cases, patients explicitly deferred decisions to the healthcare professional: ‘…*patients often say to me… “you are the expert”. I think at that stage it is absolutely acceptable*…’^21^ (p. 153). These situations reflect how patient limitations, preferences and, perhaps, ingrained power dynamics might result in clinicians leading goal setting.

### We have to build their understanding first

Initially, patients can have a limited understanding of what rehabilitation entails and the potential benefits of it, which can complicate the goal-setting process. Clinicians described how some patients struggled to see the relevance of therapy: ‘*They might think: ‘I don't see the point of this, I just want to go home and it will all be fine’ […] they don't have the insight to understand that they have to do certain things before they can achieve that overall goal’*^22^ (p. 311). To move forward, clinicians gradually worked to build understanding, including clarifying their own professional roles: ‘*It's to make sure the patient understands my role as an occupational therapist […] if they don’t really understand that, it is quite difficult to set goals which are relevant and meaningful’*^22^ (p. 311). Healthcare professionals also experienced that this education is something they do with families, in addition to patients, indicating the systemic role of education and shared understanding of goal setting in rehabilitation.

### Sometimes we are not on the same page

Goal setting could highlight conflicting perspectives between patients, families and professionals. Tensions may arise between families and clinicians in terms of views on goal setting: ‘*As a professional you have one idea and the family have another’*^22^ (p. 312), often requiring negotiation and compromise to balance family and patients’ expectations and professional judgment. Furthermore, differences in how goal setting was understood emerged within multidisciplinary teams, with one clinician noting: ‘*Consultants’ perspectives of goals are very practical […] therapists have quite a theoretical […] concept of goal setting […] nurses have something different again’*^21^ (p. 153). These examples suggest that rather than focusing solely on patient needs and wishes, clinicians often navigate multiple, sometimes competing, expectations.

### Too busy to be person-centred

Experiences described how clinicians perceive workload and service demand as limiting their engagement in meaningful, person-centred, goal setting. The fast pace of rehabilitation settings left little space for collaboration: ‘*We could do it (goal setting) better … the culture and the busyness of the ward … hampers us most’*^21^ (p. 153). There is a perceived value of person-centred and tailored care, but time pressures and workload frequently interrupt this approach: ‘*I know that I should spend time and talk to the patients and work out what is important to them […] but it's too hard […] I have five more patients to see…’*^22^ (p. 312). Goal setting in this context occurs at best hastily, and without adequate patient input, as a more person-centred approach is perceived as less time efficient. Discharge targets dominated, and limited time also hindered the involvement of families, despite their essential insights. This theme suggests that goal setting with the person in the centre risks being compromised in favour of short-term service-related targets.

### Getting to know the person behind the patient

Clinicians emphasised that truly person-centred goal setting was not simply about collecting background information but discovering what motivates each patient and how this should inform their rehabilitation. One therapist reflected, ‘*Goal exploration is key in the initial phases because it tells us what the patient's interests are […] It gives us a target and a direction’*^22^ (p. 313). Informal, conversational approaches were adopted to elicit personal goals, as one clinician recalled, ‘*I don't think I'd ask in such a [formal] way but I would do it in a more chatty sort of way […] what do you miss doing or is there anywhere you'd like to go?’*^18^ (p. 405). These reflections show that active efforts to understand the person behind the patient ensure rehabilitation goals are personally meaningful and anchored in the patient's identity, values and life story.

### Be present with the heart

This theme illustrates the relational aspects of goal setting, emphasising the need to establish trust and show a genuine presence. Clinicians’ views were that patients were unlikely to engage meaningfully without feeling accepted and valued, whilst creating a respectful space encouraged openness. Clinicians echoed the importance of fostering a therapeutic relationship, where they would be ‘*…on an equal level with the client. […] I see myself more as a supporter’*^17^ (p. 3630). Several practitioners reflected that these relational skills took time and experience to develop: ‘*Some therapists are more orientated to doing it than others – it depends on individual personalities and how much you have experienced’*^21^ (p. 151). These accounts suggest that therapeutic rapport was as crucial as clinical skill, enabling goals to emerge from a foundation of trust, empathy and emotional sensitivity. The therapeutic report was perceived as linked to the experience and personality of clinicians, which could suggest that it is seen more as a ‘soft’ skill not enhanced via training or education.

### Communication as the bridge to participation

Clinicians described adapting their approach to meet each patient's unique communication needs. As one clinician expressed, ‘*What I then do … when someone has difficulties because of cognition, communication or both […] I would say that one adjusts to the individual’*^17^ (p. 3632). Therapists employed a range of creative strategies to support participation, including visual aids, written words and simplified language. Others described starting with broad yes/no questions and narrowing them down, enabling patients to express their preferences in an accessible way. In some cases, clinicians relied on non-verbal cues for confirmation: ‘*She has been able to communicate whether she is happy or not to participate in the decisions […] by nodding or shaking her head’*^19^ (p. 166). These examples highlight the resourceful efforts clinicians make to ensure that patients are still heard despite communication barriers.

### Emotions shape the conversation

Healthcare professionals were reflecting on various emotional aspects of patient's adjustment and how this related to their own experience of goal setting. In the early phase, patients are still processing their diagnosis and loss, and healthcare professionals are aware of these needs when approaching goal-setting conversations: ‘*Addressing/acknowledging issues such as depression and grief has been an important and inevitable part of the patient's management’*^19^ (p. 167). Patients’ emotional vulnerability shaped what could be pursued, and clinicians adjusted their approach to accommodate emotional needs. In other instances, patients express frustration over therapy altogether and from not seeing the value of it: ‘*A lot of them will say, “Oh I am sick of playing these childish games, what's it doing?”*’^22^ (p. 313). This theme highlights how emotional adjustment, including grief, was met with sensitivity, and how clinicians adapted goal discussions.

### Analytical themes

Three overarching analytical themes were derived from the thematic synthesis. The three themes are directly related to, and built upon, some of the descriptive themes. Their conceptual relationships are illustrated in Figure 2.

### Who leads, who follows?

The extent to which stroke patients are involved in goal setting was described not as a fixed trait but as a dynamic process, shaped by both the patient and the professional. Some patients were highly engaged: ‘*We had a chap […] who would happily talk about goals and get a really clear steer on things…’*^21^ (p. 151), and clinicians welcomed this active or leading role as they were able to support their aspirations.

In contrast, some patients handed over control entirely to the healthcare professional. While patient passivity was often linked to communication or cognitive difficulties, some clinicians acknowledged falling into a directive role by default: ‘*I have to admit that sometimes I just do what I think is best […] but it's not like we really negotiate about goals’*^16^ (p. 875).

In multidisciplinary teams, clinicians sometimes prioritised goals that suited the service over those that reflected the individual: ‘*We kind of make the goal for the patient in a language that is acceptable to the team’*^21^ (p. 153). However, some reflected that their willingness to share power developed over time, suggesting that experience plays a role in resisting routine professional dominance ‘*The more experienced I have got, the more I am prepared to just go with what people want’*^21^ (p. 151). Overall, this theme demonstrates how both patient capacity and clinician practices bidirectionally influence the degree of collaboration in goal setting.

### Between hope and reality

Goal setting was rarely linear; instead, clinicians described the process as a careful negotiation shaped by patients’ abilities, expectations and institutional limits and cultures. Managing unrealistic hopes from patients and families was a recurring challenge, however, this extended to shifting clinicians’ own expectations: ‘*When I first started the job it was managing my expectations as a clinician, I am not going to fix people, and that was something I found hard […] and now it is a lot about managing their expectations and managing the family expectations […] set goals that mean something to them. It's far more of a negotiation’*^22^ (p. 312).

Healthcare professionals described balancing encouragement with honesty, sometimes delaying difficult conversations to protect motivation. Goal setting was perceived as an ongoing process, and goals were sometimes modified mid-process: ‘*If one realizes, that this goal isn’t achievable, or the goal is overwhelming for the client, then it could well be that one adapts the goal during the therapy’*^17^ (p. 3631). Collaborative goal setting was also disrupted by the reality of practical factors, such as privacy concerns on wards.

These accounts reveal how collaborative ideals are often undermined not by lack of willingness, but by the fragile conditions and contexts under which rehabilitation takes place. Healthcare professionals appear to experience ongoing adaptation of patients’ expectations, but also their own personal ideals of what the aim of goal setting is, often within the constraints of time pressure and competing service demands.

### Starting with the person, not the problem

Clinicians emphasised that effective rehabilitation required more than clinical expertise; it began with a human connection. This involved understanding not only what the patient could do, but who they were: ‘*To look at the client as an individual […] to find out what is important for him in his life and his personality’*^17^ (p. 3630). Empowerment was seen as a practical way of helping patients feel heard and involved. Even small acts of offering choices increased autonomy and helped foster collaboration. These efforts supported patients in ways that extended beyond engagement, enhancing their overall well-being.

Such outcomes were closely tied to the trust experienced between healthcare professionals and patients. As one clinician reflected, it was important to ‘*create mutual trust, where the people know that they are in good hands and that they are being accepted and taken seriously and appreciated, also with their deficits’*^17^ (p. 3631). This appreciation of the whole person, including their character and vulnerabilities, was crucial to shaping a strong therapeutic relationship. Some clinicians adapted to individuals intuitively, while others expressed uncertainty about how best to tailor their approach. Regardless of experience, most saw building a trusting relationship with patients as foundational to their experience of goal setting.

### Confidence in review findings

Confidence in all three analytical themes was considered moderate (see Table 3). Methodological limitations and relevance raised minor concerns across findings. Coherence was consistently rated as having no or very minor concerns. Moderate concerns were noted for adequacy in the theme ‘Starting with the person, not the problem’, due to a smaller volume of supporting data. Overall, the findings are assessed as reasonably robust and reflective of healthcare professionals’ perspectives on goal setting in stroke rehabilitation.

**Table 3.** CERQual summary of qualitative findings.

Review finding	Studies contributing to the review finding	Explanation of CERQual assessment		Overall CERQual assessment of confidence
Who leads, who follows?	Berg et al.^16^ Dörfler and Kulnik^17^ Lawler et al.^18^ Leach et al.^19^ Levack et al.^20^ Lloyd et al.^21^ Parsons et al.^22^ Rosewilliam et al.^23^	Methodological limitations	Minor concerns	Moderate confidence
		Coherence	No/very minor concerns	
		Adequacy	Minor concerns	
		Relevance	Minor concerns	
Between hope and reality	Berg et al.^16^ Dörfler and Kulnik^17^ Lawler et al.^18^ Leach et al.^19^ Levack et al.^20^ Lloyd et al.^21^ Parsons et al.^22^ Rosewilliam et al.^23^	Methodological limitations	Minor concerns	Moderate confidence
		Coherence	No/very minor concerns	
		Adequacy	Minor concerns	
		Relevance	Minor concerns	
Starting with the person, not the problem	Berg et al.^16^ Dörfler and Kulnik^17^ Lawler et al.^18^ Leach et al.^19^ Lloyd et al.^21^ Parsons et al.^22^ Rosewilliam et al.^23^	Methodological limitations	Minor concerns	Moderate confidence
		Coherence	No/very minor concerns	
		Adequace	Moderate concerns	
		Relevance	Minor concerns	

## Discussion

The synthesis identified three analytical themes that reflect the complexity of clinicians’ experiences of goal setting in stroke rehabilitation, describing how healthcare professionals negotiate between what is idealised and what is practically possible in stroke rehabilitation practices: Who leads, who follows?; Between hope and reality; and Starting with the person, not the problem.

At the centre of these findings is the suggestion that goal setting in stroke rehabilitation is often therapist-led, and not consistently person-centred or collaborative in practice. This finding aligns with research on patient accounts.^3,6,8,24^ Lloyds and colleagues^8^ synthesised the qualitative literature on the patient experience of goal setting in stroke rehabilitation, and presented four primary findings, namely: (1) Person-centred goal setting is possible but often does not occur; (2) Practitioners shape the context of goal setting; (3) Healthcare professionals need to listen to the person and know ‘who they are’ – there is a need for an individualised approach to goal setting; (4) Recovery is ongoing and unpredictable. Their review concludes that a person-centred approach to goal setting in stroke rehabilitation is possible and rewarding, but unfortunately rarely occurs in practice.

Our present review adds to previous findings by providing the perspective of healthcare professionals. From this perspective, it can be shown that healthcare professionals’ experiences describe a person-centred approach as an ideal way of approaching goal setting, and they are motivated to implement more collaborative practices, as illustrated by the sub-theme ‘Getting to know the person behind the patient’. However, their experiences outline several factors that make this way of working challenging.

First, healthcare professionals’ perspectives of goal setting involve patient factors, such as communication and cognitive impairments, emotional distress and a lack of understanding of what rehabilitation entails. Healthcare professionals describe a gradual shift in practice, with patient factors becoming more integrated into goal setting as clinicians gain experience, confidence and interpersonal skills. Prior research on facilitators to goal setting in brain injury rehabilitation has highlighted staff skills, knowledge, experience and their engagement in the process as facilitating factors.^24^ In our research, the sub-themes ‘Be present with the heart’ and ‘Communication as a bridge to participation’ capture how healthcare professionals learn to adapt to patient needs with practice and experience, including accommodations for emotional, physical or communication needs. These themes also suggest that goal setting is an ongoing process in which the healthcare professional is making continued judgements on how directive or collaborative the process can be. The experiences suggest that early goal setting may require more directive approaches.

Secondly, practical and systemic aspects of goal setting are also described in the reviewed research. Time constraints, captured within the sub-theme ‘Too busy to be person-centred’, were a prominent feature of clinicians’ experiences, consistent with prior research.^24^ Clinical guidelines recommend increasing the dose in terms of the quantity of therapy in stroke rehabilitation.^4^ If staffing is not matched to support implementation of those recommendations, the consequence could be that healthcare professionals experience being pressured to work with more patients within the same time. Personalising practice, and working collaboratively, in terms of goal setting, could be at risk of being neglected when workload increases.^25^ In the context of clinical guidelines, the present review illustrates how healthcare professionals can feel stuck between the benefits of more personalised approaches, and the lack of time to do so.

Thirdly, cultural factors were part of healthcare professional's experiences, such as how clinicians frame their own role in the process. The theme ‘Who leads, who follows?’ illustrates a default position in healthcare in which the professional is recognised as the agent leading all interactions (including assessment, diagnosis, and treatment), in accordance with a biomedical model of healthcare.^26^ Research has previously illustrated that a potential consequence of this default position, when applied in rehabilitation, is a narrative that sees the healthcare professional's role as being responsible to ‘fix’ physical impairments for a passive patient, rather than prioritising the personal meaning, values or hopes of an active patient.^23,25,27^

Healthcare professionals in stroke rehabilitation are therefore faced with the problem of being aware of the need to adhere to clinical guidelines and principles of a person-centred approach to goal setting, whilst being situated in a context where prevailing cultural norms suggest that they should lead the entire process. By examining this dynamic from the clinician's perspective, the current review shows how professional dominance is sometimes maintained unconsciously through routine, time pressure and culture, rather than intention.

Whilst methodological quality amongst the reviewed studies was high, a limitation noted was a lack of explicit statements regarding the researcher–participant relationship. This aspect is important in the present context as hierarchies in health care can be prominent and thus limit or shape participant contributions.

In terms of the present review, due to resource restrictions and the lack of translation facilities, articles in languages other than English were excluded from the review, which may have resulted in missing valuable literature and limited the inclusion of perspectives from non-English-speaking contexts. While the exclusive focus on clinicians’ perspectives provides a synthesis of an underexplored viewpoint, it reduces a range of professions in the multidisciplinary team into one collective experience. There is a risk that subtle nuances between disciplines are left out of our analysis due to our choice of including all healthcare professionals in stroke rehabilitation. As the literature is growing, it would be interesting to raise interdisciplinary differences in views of goal setting in stroke rehabilitation.

Whilst we set out to explore experiences and perspectives from healthcare professionals generally, we note that some themes could be conceptualised as barriers or facilitators to the implementation of goal setting in practice. Reflecting on this, it is possible that the clinician-led nature of the research group has influenced perspectives, with data approached from a pragmatic viewpoint, and themes structured accordingly.

Furthermore, inherent in qualitative synthesis is the limitation of the generalisability of these findings beyond the participants in the identified studies. Likewise, to stay close to participants’ own experiences and words, only primary data (i.e. direct quotes) were extracted from identified studies for the analysis. We acknowledge that there are other ways to approach qualitative meta-synthesis, including the use of researcher-derived themes, and that a more inclusive approach would have increased the amount of data available.

Methodological rigour was strengthened by involving multiple independent reviewers in key stages, including search strategy development, study selection and screening. The quality of included studies was also independently appraised by multiple raters, enhancing the reliability of the assessment. Another strength is the exclusive use of stroke-specific data, and the diversity of professional backgrounds represented across the included studies, which enhances the relevance and applicability of the review's findings to stroke rehabilitation practice.

There are implications for practice, and directions for future research, from the present findings. To enhance collaboration and person-centred goal setting in stroke rehabilitation, the perspectives of clinicians are essential to consider. Services and individual healthcare professionals implementing goal setting could build in open reflection on current habits and responses to routine demands in their practice evaluations, as these may unintentionally position them as the primary decision-makers in the context of goal setting.

Healthcare professionals may benefit from targeted training on more than the practical elements of using goal setting in rehabilitation. As such, training on goal setting beyond SMART goals would be recommended. Specifically, this training could involve how to engage patients in goal setting and rehabilitation, how to set personally meaningful goals with patients and family, and how to manage the emotional complexity of goal setting in stroke rehabilitation.

Given that organisational demands and time constraints often limit clinicians’ ability to engage in collaborative goal setting, healthcare systems should consider these systemic aspects to be barriers to the implementation of goal setting in practice. Individual healthcare professionals report a willingness to work alongside patients in accordance with guidelines on person-centred goal setting, but need the support to do so, as they also report manoeuvring a challenging reality in practice. Any training needs to be implementable and fit into a modern rehabilitation practice with busy schedules, to ensure it is not adding further to an already strained workforce.

Future research can develop and test goal-setting approaches suitable for time-limited clinical settings and explore how they can support meaningful goal setting while addressing efficiency demands. Future research could explore power dynamics in goal setting, particularly how ingrained institutional norms shape clinician–patient hierarchies, influence collaboration and lead clinicians to default to control while reinforcing patients’ views of clinicians as the only experts. Additionally, future research should explore how clinicians balance intuitive, relational approaches with more structured goal-setting methods, and whether targeted training can support this integration without diminishing empathy. Understanding how to combine emotional sensitivity with clarity and consistency could inform training programmes that help clinicians move beyond standardised practice, build deeper therapeutic relationships, and support less experienced or less confident practitioners.

In conclusion, this systematic review provides a synthesis of qualitative research on healthcare professionals’ experiences of goal setting, revealing the tension between striving for person-centred care and working within real-world constraints. Clinicians navigate emotional, relational and systemic challenges of goal setting in practice. While collaboration is widely encouraged, it is often constrained by institutional pressures, ingrained hierarchies and patient factors. More experienced clinicians, or those with strong relational skills, demonstrated a clear commitment to person-centred care, adapting flexibly to support individual needs, build therapeutic relationships and promote autonomy. These findings underscore the need for systemic support and targeted training to enable healthcare professionals to engage in meaningful goal setting in time-limited environments.

**Clinical message**

- Healthcare professionals experience goal setting as a dynamic ongoing process.
- Relational, patient and service-level challenges are perceived as impacting meaningful goal setting.
- Training for healthcare professionals could go beyond SMART goals, and target patient engagement, personally meaningful goal setting and managing the emotional complexity of goal setting in stroke rehabilitation.

## Supplemental Material

sj-docx-1-cre-10.1177_02692155261446313 - Supplemental material for Goal setting in stroke rehabilitation: A systematic review and qualitative synthesis of the experiences and perspectives of healthcare professionals Supplemental material, sj-docx-1-cre-10.1177_02692155261446313 for Goal setting in stroke rehabilitation: A systematic review and qualitative synthesis of the experiences and perspectives of healthcare professionals by Anna Fišerová, Šarlota Duchoňová, Hannah Hafiz, Martina Hagarová, Brodie Morton, Helena Tessmann, Christopher D. Graham and Nils Rickardsson in Clinical Rehabilitation

## Data Availability

All data underpinning this publication are openly available from the University of Strathclyde KnowledgeBase at https://doi.org/10.15129/3103382b-bb3e-49e8-9356-cd86c6e86e5d.
