# Supplementary material for: Goal setting in stroke rehabilitation: A systematic review and qualitative synthesis of the experiences and perspectives of healthcare professionals
Source: Clin Rehabil. 2026 May 2;40(11):1517–32. doi: 10.1177/02692155261446313 (PMC13736664; doi:10.1177/02692155261446313)
Supplement: Supplementary file 1 [file sj-docx-1-cre-10.1177_02692155261446313.docx]

**Supplementary files**

**Supplementary Table 1**

*The SPIDER tool applied to search terms*^12^

| SPIDER components | Search terms |
| --- | --- |
| S | "multidisciplinary team*" OR "healthcare professional*" OR "clinician*" OR "speech therapist*" OR "psychologist*" OR "language therapist*" OR "physiotherapist*" OR "occupational therapist*" OR "nurse*" OR "practitioner*" OR "HCP" OR "HCPs" OR "consultant physician*" OR "social worker*" OR "rehabilitation assistant*" |
| PI | AND "stroke*" OR "stroke rehab*" OR "ischemia*" OR "cerebrovascular accident*" OR "CVA*" OR "haemorrhagic*" |
| PI | AND "goal setting*" OR "goal planning*" OR “goal*” |
| E | AND “view*” OR “perspective*” OR “experience*” OR “opinion*” OR “observation*” OR “understand*” OR “insight*” OR “perce*” OR “belie*” OR “attitude*” |

**Supplementary Table 2**

*The SPIDER tool applied inclusion and exclusion Criteria*

| SPIDER components | Inclusion Criteria | Exclusion Criteria |
| --- | --- | --- |
| S | Participants include healthcare professionals involved in stroke care. | Quotes cannot be separated from those of other participant groups. |
| PI | The study explores goal setting within stroke rehabilitation. | The study does not mention goal setting or only refers to it incidentally, without meaningful quotes. |
| D | The methodology includes qualitative data collection (e.g., interviews, focus groups), if a mixed-method study, the qualitative data are clearly identifiable and separable. | The qualitative content is too sparse or unclear to extract meaningful data (e.g., goal setting mentioned but no insights into experiences or views). |
| E | The study reports on healthcare professionals’ experiences or perceptions. | Study is not focused on stroke (e.g., TBI, general neurorehabilitation without stroke-specific data). |
| R | The study is peer-reviewed primary research and published in English. | The study is a review article, conference abstract, or grey literature. |

**Supplementary Table 3**

*Summary of descriptive themes across individual studies*

| Themes | Study | | | | | | | |
| --- | --- | --- | --- | --- | --- | --- | --- | --- |
|  | Berg et al.^16^ | Dörfler and Kulnik^17^ | Lawler et al.^18^ | Leach et al.^19^ | Levack et al.^20^ | Lloyd et al.^21^ | Parsons et al.^22^ | Rosewilliam et al.^23^ |
| When they come in with a goal already | ✔ | **X** | **X** | **X** | ✔ | ✔ | ✔ | **X** |
| Leading the way, for now | ✔ | ✔ | ✔ | ✔ | **X** | ✔ | ✔ | ✔ |
| We have to build their understanding first | ✔ | ✔ | ✔ | ✔ | **X** | **X** | ✔ | **X** |
| Sometimes we’re not on the same page | ✔ | ✔ | ✔ | **X** | **X** | ✔ | ✔ | **X** |
| Emotions shape the conversation | **X** | **X** | ✔ | ✔ | ✔ | **X** | ✔ | ✔ |
| Too busy to be person-centred | **X** | ✔ | **X** | ✔ | **X** | ✔ | ✔ | ✔ |
| Getting to know the person behind the patient | ✔ | ✔ | ✔ | ✔ | **X** | **X** | ✔ | **X** |
| Be present with the heart | ✔ | ✔ | ✔ | **X** | **X** | ✔ | ✔ | **X** |
| Communication as a bridge to participation | ✔ | ✔ | **X** | ✔ | **X** | **X** | **X** | **X** |

*Note.* ✔ = Yes. **X** = No.

**Supplementary Table 4**

*Additional supporting quotations for identified themes*

| Theme | Additional supporting quotations |
| --- | --- |
| When they come in with a goal already | *“Pretty straightforward really, he pretty much tells you what he wants […] before you even get to asking him really, he knows exactly what he wants to achieve. . . he has goals that you can directly take and make into treatments”*^20^(p. 211). |
| Leading the way, for now | *“The biggest challenges come with the most severe aphasias, where the client barely can say yes and no. And then it is expected that we come up with measurable goals, and written descriptions where . . . I make the goal, and at best I get a confirmation that the client agrees”*^16^ (p. 875).  *“ […] You might have to go through a period of … therapist-led goals”*^21^(p. 153). |
| We have to build their understanding first | *“After education of family [...], their expectations were changed given all the information, history and stroke recovery outcomes”*^19^(p. 164).  *“Sometimes one must guide them there, that they realize, that it’s necessary to work on something […] maybe it first needs several single sessions, to somehow gently try and show them that they have deficits and to let them become aware of that, so to say. And then one can say, okay, let us formulate the goals once again.”*^17^(p. 3632). |
| Sometimes we are not on the same page | *“It is practically impossible to pursue a goal which the partner didn’t find important or even openly rejected”*^17^(p. 3633).  *“… It often ends up being a bit of negotiation … (with) the patient … having the higher expectations, the therapist maybe having the lower, hopefully slightly more realistic, expectations, and then … negotiating and meeting maybe somewhere in the middle”*^21^(p. 153). |
| Emotions shape the conversation | *“If you have got someone who is low in mood and is struggling with their diagnosis, the way their futures looking, I think sometimes you have to read a bit carefully and maybe, you know, thinking about making things as achievable as possible”*^22^(p. 311).  *“I think you would play it by ear with individual patients because you could not upset them or dash their hopes… you didn't want to upset them, you know, the nurse doesn't think I'm going to get anywhere, then there's no point in me trying, so you had to keep encouraging”*^18^(p. 406). |
| Getting to know the person behind the patient | *“If you are not working towards something that the patient is interested in then invariably they wouldn't have the motivation to participate in whatever programme we set, so we always try to build a rapport with them right from the outset. Find out what it is they want to work towards,and try and tweak your goals together, throughout the process, just to keep them engaged and motivated.”*^22^(p. 313).  *“[…] you'd ask them what they'd done in the past and would they like to do that again?[…]”*^18^(p. 405). |
| Be present with the heart | *“It is really important to be present with the heart […] to listen with the heart”*^17^(p. 3632).  *“I wanted them to become more open so I could gain their confidence so they could express their difficulties”*^18^(p. 406). |
| Communication as the bridge to participation | *“I outlined in simple verbal, written words and pictures things that we could work on and she was able to identify (point to), after this, what things she wanted to focus on […]”*^19^ (p. 166).  *“I was able to determine patient goals by phrasing questions with yes/no responses. I started with large goals e.g. do you want to be able to walk? And then narrowed these down e.g.do you want to be able to walk without help?”*^19^  (p. 166). |
| Too busy to be person-centred | *“We're probably setting the goals for the patient, in a rush the night before”*^22^(p. 313)  *“…it is rehab but it’s just the fact that we have less time to do rehab because you have so many new patients to see. You’re sending out people, discharging people quickly…”*^23^(p. 515). |
| Who leads, who follows? | “*They often see us as the expert and want us to tell them what to do […] It is hard to get them to identify what they want in that situation*”^22^(p. 312). |
| Between hope and reality | “*At that stage I couldn't tell him. I think I thought in my own mind that he wouldn't drive again but at that stage if I had told him it would have flattened him… I just said to him, ‘It’s early days… at this stage I can’t tell whether you will…*’”^18^(p. 406).  “*Sometimes it is a bit difficult, like with the four bedded bays and when you’re talking to a patient it’s not very private… Sometimes, the patient can get a bit upset. You don’t want to speak on sensitive issue, because that can be awkward…*”^23^(p. 516). |
| Starting with the person, not the problem | “*It is more about… trying to empower people to have a say in what they want*”^21^(p. 151).  *“Quality of life increases, especially when the patient can decide himself where he wants help and where not*”^17^(p. 3632). |
